# Do patients with chronic unilateral orofacial pain due to a temporomandibular disorder show increased attending to somatosensory input at the painful side of the jaw?

**DOI:** 10.7717/peerj.4310

**Published:** 2018-01-24

**Authors:** Stefaan Van Damme, Charlotte Vanden Bulcke, Linda Van Den Berghe, Louise Poppe, Geert Crombez

**Affiliations:** 1 Department of Experimental-Clinical and Health Psychology, Ghent University, Ghent, Belgium; 2 Department of Dentistry, Ghent University Hospital, Ghent, Belgium; 3 Department of Movement and Sport Sciences, Ghent University, Ghent, Belgium; 4 Center for Pain Research, University of Bath, Bath, United Kingdom

**Keywords:** Chronic pain, Orofacial pain, TMD, Somatosensory processing, Hypervigilance, Attentional bias, Spatial attention, Temporal order judgement

## Abstract

**Background:**

Patients with chronic orofacial pain due to temporomandibular disorders (TMD) display alterations in somatosensory processing at the jaw, such as amplified perception of tactile stimuli, but the underlying mechanisms remain unclear. This study investigated one possible explanation, namely hypervigilance, and tested if TMD patients with unilateral pain showed increased attending to somatosensory input at the painful side of the jaw.

**Methods:**

TMD patients with chronic unilateral orofacial pain (*n* = 20) and matched healthy volunteers (*n* = 20) performed a temporal order judgment (TOJ) task indicated which one of two tactile stimuli, presented on each side of the jaw, they had perceived first. TOJ methodology allows examining spatial bias in somatosensory processing speed. Furthermore, after each block of trials, the participants rated the perceived intensity of tactile stimuli separately for both sides of the jaw. Finally, questionnaires assessing pain catastrophizing, fear-avoidance beliefs, and pain vigilance, were completed.

**Results:**

TMD patients tended to perceive tactile stimuli at the painful jaw side as occurring earlier in time than stimuli at the non-painful side but this effect did not reach conventional levels of significance (*p* = .07). In the control group, tactile stimuli were perceived as occurring simultaneously. Secondary analyses indicated that the magnitude of spatial bias in the TMD group is positively associated with the extent of fear-avoidance beliefs. Overall, intensity ratings of tactile stimuli were significantly higher in the TMD group than in the control group, but there was no significant difference between the painful and non-painful jaw side in the TMD patients.

**Discussion:**

The hypothesis that TMD patients with chronic unilateral orofacial pain preferentially attend to somatosensory information at the painful side of the jaw was not statistically supported, although lack of power could not be ruled out as a reason for this. The findings are discussed within recent theories of pain-related attention.

## Introduction

Temporomandibular disorders (TMD) represent a group of highly prevalent and disabling conditions characterized by problems and dysfunctions in the jaw muscles and/or in the temporomandibular joint, with chronic orofacial pain as one of the typical features (Turner et al., 2001). TMD are poorly understood and difficult to treat, and likely to be affected by multiple factors (Suvinen et al., 2005). It has been proposed that abnormalities in somatosensory processing may play a role (Nebel et al., 2010). For instance, it has been found that TMD patients, relative to healthy controls, report tactile stimuli at the jaw as more intense (Ayesh, Jensen & Svensson, 2007) and display enhanced cortical responses to such stimuli (Alonso et al., 2010). However, insight in the exact nature and underlying mechanisms of somatosensory amplification in TMD is largely missing. For example, although TMD-related orofacial pain is more often unilateral than bilateral, studies investigating somatosensory amplification have rarely differentiated between the painful and non-painful side of the jaw (Ayesh, Jensen & Svensson, 2007; Kothari et al., 2015).

One possible source of amplified somatosensory perception in chronic pain patients is hypervigilance, that is, the process of habitually attending to pain-related information such as bodily cues (Chapman, 1978; Crombez, Van Damme & Eccleston, 2005; Hollins et al., 2009; McDermid, Rollman & McCain, 1996). Hypervigilance is thought to emerge as a result of the experience of threat (Eccleston & Crombez, 1999; Van Damme et al., 2010), for instance, in patients with high levels of pain-related fear (Vlaeyen & Linton, 2000). It has been proposed that an important feature of hypervigilance is increased attending to somatosensory input at pain-relevant body locations (Van Damme et al., 2016). This idea has been extensively investigated in a series of studies in healthy volunteers indicating that experimentally induced threat of pain on one specific location of the body resulted in increased attending to somatosensory stimuli at that body location relative to other areas (Crombez et al., 1998; Durnez & Damme, 2015; Vanden Bulcke et al., 2013; Vanden Bulcke et al., 2014; Vanden Bulcke et al., 2015; Van Hulle et al., 2015; but see Durnez & Van Damme, 2016, for a failed replication). Applied to unilateral TMD pain, one would expect enhanced attending to somatosensory information *specifically* at the painful side of the jaw. However, studies investigating location-specific attention biases in clinical samples, including TMD, are currently lacking. Understanding the presence and nature of somatosensory hypervigilance in TMD may help us determine new treatment for these patients.

The present study aimed to investigate whether TMD patients with unilateral orofacial pain show a bias in somatosensory attending to the painful jaw side. For this purpose, we used a Temporal Order Judgment (TOJ) paradigm (Piéron, 1952), assessing the order in which individuals perceive pairs of stimuli. The paradigm has been typically used to investigate the law of prior entry (Titchener, 1908), stating that attended stimuli come into consciousness earlier than unattended stimuli (see Shore et al., 2005; Spence, Shore & Klein, 2001). More recently, TOJ tasks with tactile stimuli have been used to demonstrate biases in somatosensory attention to or away from pain-related body locations (Moseley, Gallace & Spence, 2009; Reid et al., 2016; Van Damme et al., 2009; Vanden Bulcke et al., 2013). In the current study, a sample of TMD patients with unilateral chronic orofacial pain and a healthy control sample indicated which one of two tactile stimuli, presented at either side of the jaw, they had perceived first. As the primary hypothesis, we tested if TMD patients would be preferentially attending to the painful side of their jaw, which should be reflected by a tendency to perceive tactile stimuli at the painful jaw side as occurring earlier than tactile stimuli at the non-painful jaw side. No difference in tactile processing between both sides of the jaw was expected in the control group. In addition, we conducted secondary analyses in the TMD sample to examine correlations between spatial bias in tactile processing and a range of self-reported pain cognitions, including pain-related fear, catastrophizing, and vigilance.

## Materials & Methods

### Participants

TMD patients were a convenience sample recruited in the Department of Dentistry at the Ghent University Hospital. Inclusion criteria included having chronic (longer than three months) unilateral orofacial pain due to a temporomandibular disorder assessed by the dentist, having an age between 18 and 65 years, and being Dutch speaking. Exclusion criteria were the presence of a severe psychiatric/neurological condition, and the presence of non-TMD related chronic pain problems. Based upon power calculations (see further), we recruited 20 TMD patients and 20 healthy controls. Potential participants were informed about the possibility of participating by means of a flyer and information given by the clinician. When they agreed to participate, they received a phone call from the researcher providing more detailed information about the study. Twenty-one patients agreed to participate in the experiment. It was not registered if they had participated in previous studies. One woman (40 years, right-handed) had to be excluded because she reported chronic widespread pain (fibromyalgia). The age of the remaining 20 TMD patients (17 females) was 36.8 years (SD = 11.6, range = 22–59 years). Mean duration of pain was about 14 months (SD = 11.3 months, range 4–36). Ten patients reported pain at the left jaw, the other ten had pain at the right jaw. The majority of the sample (70%) had not received treatment for their TMD at the time of testing. An overview of the demographic characteristics is provided in Table 1.

**Table 1:** Demographic characteristics of the patient and control group.

	TMD patients		Control group	
	M ± SD	N (%)	M ± SD	N (%)
Men		3 (15%)		2 (10%)
Women		17 (85%)		18 (90%)
Age (in years)	36.8 ± 11.66 (range 22–59)		36.9 ± 13.90 (range 20–63)	
Family situation				
Single		4 (20%)		12 (60%)
Living together		5 (25%)		4 (20%)
Married		8 (40%)		4 (20%)
Widow(er)		3 (15%)		0
Educational level				
Primary education		0		0
Lower secondary education		2 (10%)		1 (5%)
Higher secondary education		6 (30%)		8 (40%)
Higher education		4 (20%)		4 (20%)
Higher education: university		8 (40%)		7 (35%)
Profession				
Housemen/housewife		1 (5%)		1 (5%)
Laborer		2 (10%)		0
Employee		10 (50%)		10 (50%)
Professional		0		0
Senior manager		0		1 (5%)
Disabled		3 (15%)		1 (5%)
Student		4 (20%)		5 (25%)
Job seeker		0		2 (10%)

The control group was recruited from an existing database, consisting of individuals from the general population who had expressed interest to participate in scientific studies of the Ghent Health Psychology Research Group, by registering at a website. Potential participants were selected with the aim of obtaining a control group matched at group level with the TMD group for age, gender and educational level. Inclusion criteria for the control participants were being Dutch speaking and having an age between 18 and 65 years. Exclusion criteria were presence of chronic pain complaints, presence of current orofacial pain, and presence of neurological/psychiatric conditions. Eligible participants were contacted and provided with information about the study. Twenty-one healthy volunteers were willing to participate. One man (23 years, right-handed) was excluded for further analysis due to not attaining the requested performance criteria during the task (see TOJ data handling). The age of the remaining 20 participants (18 females and two males) was 36.9 years (range 20–63 years; SD = 13.9). An overview of the demographic characteristics is provided in Table 1. Statistical analyses showed no differences between groups in gender, *χ*^2^(1) = 0.23, *p* = .633, mean age, *t*(38) =  − 0.03, *p* = .980, and educational level, *χ*^2^(3) = 0.69, *p* = .877.

The study was approved by the Medical Ethical Committee of the Ghent University Hospital (B670201213538). At the end of the experiment, all participants received 25 Euro as reimbursement for their expenses. The experimental session lasted for approximately 1 h and a half.

### Apparatus and materials

Our tactile stimulation procedure was similar as what has been described in previous studies (e.g., Vanden Bulcke et al., 2013). We presented tactile stimuli at both sides of the jaw with a duration of 10 ms duration and a frequency of 200 Hz, using two resonant-type tactors (C-2 TACTOR; Engineering Acoustics, Inc., Casselberry, FL, USA; http://www.eaiinfo.com/) consisting of a housing of 3.05 cm diameter and 0.79 cm high, and a skin contactor of 0.76 cm diameter. In order to control for differences in tactile sensitivity between both sides of the jaw, subjective stimulus intensity was individually calibrated and matched between jaw sides before the experiment using a double random staircase procedure (see Levitt, 1971; Weinstein, 1968). In a first phase, participants rated 24 stimuli presented on the left side of the jaw relative to a reference stimulus, which was defined as the maximum intensity (power = 0.21 Watt) on a 5-point Likert scale ranging from 1 (‘no sensation’) to 5 (‘maximum intensity’). The intensity that elicited an averaged rating of 3 was used as the stimulus intensity for the left jaw side, and was the reference stimulus for the second phase. In the second phase, participants rated 24 stimuli on the right jaw side relative to the reference stimulus on the left jaw side on a 5-point Likert scale (1 = ‘much weaker’, 2 = ‘weaker’, 3 = ‘equally strong’, 4 = ‘stronger’, 5 = ‘much stronger’). The intensity that elicited an averaged rating of 3 was used as the intensity of the stimulus at the right jaw side.

The task was programmed and controlled by the INQUISIT Millisecond software package (Inquisit 3.0, Millisecond Software LLC, Seattle, WA, USA; http://www.millisecond.com/) on a laptop (Dell Vostro 3550).

### TOJ paradigm

The tactile TOJ paradigm is considered a particularly suitable methodology to assess spatial biases in tactile processing (Shore et al., 2005), and has also been used in the context of pain (e.g., Moseley, Gallace & Spence, 2009; Vanden Bulcke et al., 2014; but see (Filbrich et al., 2016; Van Damme et al., 2016), for some methodological considerations and challenges). Specifically, two tactile stimuli were administered, one on either side of the jaw, separated by one of 10 randomly assigned stimulus onset asynchronies (SOAs) between −200 to +200 ms (−200, −90, −55, −30, −10, +10, +30, +55, +60, +200 ms). Each trial was preceded by the presentation of a fixation cross (2,000 ms) in the middle of the screen. The participants reported verbally at which side of the jaw they first had perceived the tactile stimulus. The experimenter registered their responses using a keyboard. Note that the SOAs in this study were modified (made larger) as compared with previous studies with undergraduate students (−120, −60, −30, −15, −5, +5, +15, +30, +60, +120; see Vanden Bulcke et al., 2013), because pilot testing with TMD patients and adults from the general population indicated that with these values too many participants would not have been able to meet the required performance criterion.

### Self-report measures

A general questionnaire was used to collect sociodemographic data (gender, age, education level, profession, family situation), presence of medical/psychological problems, and use of pain medication. In the TMD group, this questionnaire also assessed which side of the jaw was painful, duration of the TMD problem, current treatment status (whether they had received treatment for their TMD or not), and presence of other chronic pain complaints. Several validated self-report questionnaires measuring pain and pain-related cognitions were used. Internal consistency (Cronbach’s alpha) was acceptable for all these instruments (see Table 2).

**Table 2:** Means (and standard deviations) and Cronbach’s alpha of self-report questionnaires in both groups.

	TMD group		Control group	
	M (SD)	Alpha	M (SD)	Alpha
MPI: pain severity	1.9 (1.1)	.70	0.9 (0.9)	.69
MPI: interference	2.0 (1.9)	.96	0.8 (1.0)	.95
TSK-TMD: total	24.8 (6.4)	.81	–	–
TSK-TMD: activity avoidance	16.5 (4.7)	.78	–	–
TSK-TMD: somatic focus	8.3 (2.6)	.63	–	–
PVAQ: total	45.1 (15.5)	.94	32.3 (13.1)	.85
PVAQ: attention to pain	26.0 (9.8)	.91	16.3 (7.4)	.77
PVAQ: attention to changes in pain	19.1 (6.8)	.88	16.1 (8.9)	.94
PCS: total	18.4 (13.0)	.96	16.3 (11.0)	.93
PCS: rumination	6.7 (4.2)	.86	6.9 (4.2)	.90
PCS: magnification	3.8 (3.3)	.85	3.5 (2.7)	.73
PCS: helplessness	7.9 (6.2)	.93	6.0 (5.3)	.89

Pain severity and pain interference were assessed by means of the Dutch version of the Multidimensional Pain Inventory (MPI-DV; Kerns, Turk & Rudy, 1985). This questionnaire consists of 28 items rated on a 7-point scale measuring severity of the pain problem (e.g., ‘Rate the level of your pain at the present moment’), interference with daily-life activities (e.g., ‘In general, how much does your pain interfere with your day-to-day activities?’), perceived control (e.g., ‘During the past week how much control do you feel you have had over your life?’), affective anxiety (e.g., ‘During the past week how irritable have you been?’) and social support (e.g., ‘How supportive or helpful is your significant other to you in your relation to your pain?’). Only the first two subscales were included.

The Tampa Scale for Kinesiophobia for Temporomandibular Disorders (TSK-TMD; Visscher et al., 2010) consists of 12 items that need to be rated on a 4-point numerical rating scale (1 = “strongly disagree”, 4 = “strongly agree”). The subscale ‘Activity Avoidance’ (e.g., ‘I am afraid that I might injure myself if I move my jaw’) consists of seven items, whereas the other subscale ‘Somatic focus’ (e.g., ‘My jaw is telling me that something is seriously wrong with it”) consists of five items. The TSK-TMD has been shown to be valid and reliable (Visscher et al., 2010).

The Pain Catastrophizing Scale (PCS; Sullivan, Bischop & Pivik, 1995) is a 13-item scale to assess catastrophic thoughts about pain in both non-clinical and clinical populations. Participants are asked to reflect on past painful experiences and to indicate the degree to which they experienced each of the 13 thoughts or feelings during pain on a 5-point scale from 0 (not at all) to 4 (all the time). The PCS consists of three subscales: rumination (e.g., ‘I keep thinking about how much it hurts’), magnification (e.g., ‘I become afraid that the pain may get worse’), and helplessness (e.g., ‘There’s nothing I can do to reduce the intensity of the pain’). The Dutch version of the PCS has been shown to be valid and reliable in both healthy populations and chronic pain patients (Van Damme et al., 2002).

The Pain Vigilance and Awareness Questionnaire (PVAQ; McCracken, 1997) contains 16 items rated on a 6-point scale measuring self-reported vigilance for pain sensations (1 = “never”, 5 = “always”). The PVAQ consists of two subscales: attention to pain (e.g., ‘I focus on sensations of pain’), and attention to changes in pain (e.g., ‘I am quickly to notice changes in pain intensity’). The Dutch version of the PVAQ has been shown to be valid and reliable in both healthy populations and chronic pain patients (Roelofs et al., 2002; Roelofs et al., 2003).

After each test phase, the participants were asked to rate the perceived intensity of the tactile stimuli (‘How intense did you experience the stimuli on your left/right jaw?’) on an eleven-point numerical rating scale (anchored 0 = not at all and 10 = very strongly). This was included to check for potential sensitization or habituation effects.

### Procedure

Upon arrival at the laboratory, participants were informed about the experimental procedures and provided written informed consent. After this, they completed a series of questionnaires (see self-report measures) in the following order: general questionnaire, MPI-DV, TSK-TMD (only TMD patients), PCS, and PVAQ. Next, they were seated in front of the experimental apparatus. Their forearms were positioned symmetrically on the table. The tactors were placed in the middle of the superficial head of the masseter muscle of each side of the jaw. The participants were informed that they had to decide on each trial which stimulus had been presented first. The accuracy of participants’ responses was emphasized, rather than the speed. Participants wore noise-cancelling headphones (PXC 350 Sennheiser) to prevent any interference from environment noise. Following this, the session began with a practice block of twenty trials (two trials per SOA). Next, four blocks of 70 trials (seven trials per SOA) were presented. The participants were not given any feedback about their performance. After each block, they were asked to rate perceived intensity of tactile stimuli at both jaws. These data were not used to adapt stimulus intensity in case of left–right differences. The participants also completed several single rating scales gauging concentration, effort, attention, and fatigue (not reported).

### Data-analyses

#### TOJ data handling

The analyses were based upon procedures that have been commonly described in the literature (Shore et al., 2005; Spence, Shore & Klein, 2001). The proportions of ‘left-jaw-first’ and ‘right-jaw-first’ responses for all trials at each SOA, were converted into the corresponding z-scores using a standardized cumulative normal distribution. The best-fitting straight line was computed for each participant and the derived slope and intercept values were used to compute the just noticeable difference (JND) and the point of subjective simultaneity (PSS). JND is monotonically related to the slope of the psychometric function and indicates the (virtual) interval needed to achieve 75% correct performance, and as such provides a standardized measure of the sensitivity of participants’ temporal perception.

PSS refers to the (virtual) SOA at which participants report the two events (in this case: right jaw first and left jaw first) equally often. The PSS is computed as the opposite of the intercept divided by the slope from the best-fitting straight line. It is customary to code SOAs so that negative values indicate that the *test* stimulus was presented before the *reference* stimulus. In the TMD sample, we regard stimuli at the painful jaw side as test stimuli, while stimuli at the non-painful jaw side are labeled as reference stimuli. In the remainder of the manuscript, positive SOAs refer to trials in which the stimulus at the non-painful jaw side preceded the stimulus at the painful jaw side. When interpreting effects on the PSS measurement, it is thus important to keep in mind that positive values indicate that stimuli stemming from the non-painful jaw side should be presented *before* stimuli originating from the painful jaw side for both to be perceived as simultaneously occurring. Correspondingly, positive PSS values indicate that tactile input at the painful jaw side is prioritized over tactile input at the non-painful jaw side. In the control group, we made the (arbitrary) choice to consider stimuli at the right jaw side as test stimuli, while stimuli at the left jaw side were reference stimuli. Here, positive values indicate that stimuli stemming from the left jaw side should be presented *before* stimuli originating from the right jaw side for both to be perceived as simultaneously occurring. Correspondingly, positive PSS values indicate that tactile input at the right jaw side is prioritized over tactile input at the left jaw side.

In line with Spence, Shore & Klein (2001), we excluded participants (from both groups) from statistical analysis when any of their PSS values was larger than the highest SOA (±200 ms). Participants were also excluded when less than 80% of their responses on trials with the largest SOA (±200 ms) was incorrect. Note that Spence, Shore & Klein (2001) originally used a cut-off of 90%, but that we have previously decided to deviate from this because much more participants would have to be excluded than what has been typically reported by Spence, Shore & Klein (2001), even in undergraduate students (see Vanden Bulcke et al., 2013; Vanden Bulcke et al., 2014). As a result, one participant of the control group (male, right-handed) was removed from data analysis.

#### TOJ hypothesis testing

We tested in each group whether the obtained mean PSS value was significantly different from the actual point of simultaneity (virtual SOA of 0 ms) using one-sample *t*-tests. Based upon the study by Vanden Bulcke et al. (2013), reporting an effect size of 0.70 for enhanced tactile processing at a hand threatened by experimental pain (relative to the other hand) in healthy volunteers, the current study was powered to detect an effect size of 0.70 with 80% power at alpha <.05 for a one-sample *t*-test. As a result, at least 19 patients were needed. As an additional analysis, we compared the mean PSS between the TMD group and the control group. However, it should be noted that the meaning of these values is different for these groups (see TOJ data handling). In the control group a positive PSS simply means that tactile input at the right jaw side is prioritized over tactile input at the left jaw side. In the TMD sample a positive PSS means that tactile input at the painful jaw side (irrespective of whether this is left or right) is prioritized over tactile input at the non-painful jaw side. As a secondary, exploratory, analysis, we tested within the TMD group whether the PSS was different between those that had pain at the left jaw side (*N* = 10) and those that had pain at the right jaw side (*N* = 10), using an independent-samples *t*-test. We had no specific hypotheses regarding the JND.

#### Perceived intensity of tactile stimuli

As a validity check, to prevent explanations of TOJ effects in terms of differences in subjective tactile intensity, we compared perceived intensity (average across the four blocks) between the painful and the non-painful side of the jaw in the TMD group, and between the left and the right side of the jaw in the control group, using paired-samples *t*-tests. As an exploratory analysis, we also compared average perceived tactile intensity between the TMD and the control groups, using an independent samples *t*-test.

#### Questionnaires

For the TSK-TMD we only provide descriptive statistics for the TMD group. For all other questionnaires (MPI, PCS, PVAQ) we compared scores between TMD patients and controls, using independent-samples *t*-tests. As a secondary analysis, we calculated Pearson correlations in the TMD group only, to examine possible associations between questionnaire scores and the behavior measure of spatial bias (PSS).

In all analyses significance level was set at *p* <.05. For ease of comparison with the norms of Cohen (1988), we calculated effect sizes for independent samples using the formula of Dunlap et al. (1996). We determined whether Cohen’s d was small (≤0.20), medium (≤0.50), or large (≤0.80). We also report the 95% confidence intervals (95% CI) of the effect sizes.

## Results

### Sample characteristics

Table 2 represents the average scores and standard deviations for the self-report questionnaires in the TMD group and the control group (except for TSK-TMD). Scores on the MPI were quite similar as those found in a large (*N* = 491) sample of TMD patients (Reissman et al., 2008), except for the pain severity subscale, which was almost 1 standard deviation lower in the current sample. The mean score for the TSK-TMD (*M* = 24.75, SD = 6.44, range 14–40) was similar as the score obtained in a large sample of TMD patients (*N* = 301, *M* = 24.2, SD = 6.9) (Visscher et al., 2010).

With regard to the MPI, independent samples *t*-tests revealed that the TMD group had significantly higher scores as compared to the control group (pain severity: *t*(38) = 3.33, *p* = .002; pain interference: *t*(38) = 2.67, *p* = .011). For the PVAQ, independent samples *t-* tests revealed that the TMD group had significantly higher scores as compared to the control group, *t*(38) = 2.62, *p* = .01). No significant differences between both groups were found on the Pain Catastrophizing Scale (*t*(38) = 0.55, *p* = 0.58).

### Tactile intensity and validity check

No significant difference in perceived intensity between tactile stimuli presented on the painful side (*M* = 5.56, SD = 2.02) and tactile stimuli presented on the non-painful side (*M* = 5.31, SD = 2.27) was found in TMD patients, *t*(19) = 0.80, *p* = 0.44. Perceived intensity of tactile stimuli (across sides and blocks) was significantly higher in the TMD group (*M* = 5.44, SD = 2.03) than in the control group (*M* = 3.98, SD = 1.99), *t*(38) = 2.29, *p* = .028 (*d* = 0.71 (95% CI [0.07–1.35]). When considering the results on perceived tactile intensity it is important to note that the *actual* intensity of administered tactile stimuli was not different between the TMD group (*M* = 22.78, SD = 3.47) and the control group (*M* = 21.93, SD = 3.10), *t*(38) = 0.82, *p* = .419, and that in the TMD group there was no significant difference in actual tactile intensity between the painful (*M* = 23.05, SD = 4.85) and the non-painful side of the jaw (*M* = 22.50, SD = 3.86), *t*(19) = 0.46, *p* = 0.651. Possible TOJ effects in TMD patients thus could not be due to differences in tactile stimulation between the painful and non-painful side of the jaw.

**Table 3:** Correlations between self-report measures and the TOJ outcome measure (PSS) for the TMD group.

	*1*	2	3	4	5	6	7	8	9	10
1. PSS	–									
2. TSK-TMD (TOT)	*.31*	–								
3. TSK-TMD (AA)	*.15*	.94^c^	–							
4. TSK-TMD (SF)	***.50***^a^	.78^c^	.53^a^	–						
5. PVAQ (TOT)	*.17*	.29	.10	.55^a^	–					
6. PVAQ (PAIN)	*.16*	.27	.05	.59^b^	.95^c^	–				
7. PVAQ (CHANGES)	*.15*	.27	.14	.41	.90^c^	.73^c^	–			
8. PCS (TOT)	*.09*	.43	.45^a^	.26	.40	.37	.38	–		
9. PCS (RUM)	*−.13*	.27	.31	.10	.44	.42	.39	.93^c^	–	
10. PCS (MAG)	*.29*	.50^a^	.47^a^	.38	.37	.36	.32	.93^c^	.76^c^	–
11. PCS (HELP)	*.11*	.46^a^	.48^a^	.27	.35	.31	.36	.98^c^	.87^c^	.90^c^

**Notes.**

a *p* < .05.

b *p* < .01.

c *p* < .001.

TOT total score AA Activity Avoidance SF Somatic Focus PAIN Attention to pain CHANGES Attention to pain changes RUM rumination MAG magnification HELP Helplessness

### Hypothesis testing

Analysis of the PSS values in the TMD group showed that tactile stimuli delivered to the non-painful side of the jaw had to be delivered before tactile stimuli at the painful side, for both stimuli to be perceived as occurring at the same time (*M* = 17.59, SD = 41.51), but the mean difference with the actual point of simultaneity (0 ms) was not significant, *t*(19) = 1.90, *p* = .07 (*d* = 0.42, (95% CI [−0.19–1.03])). There was no significant difference in PSS values between patients with left jaw pain (*M* = 7.15, SD = 41.89) and patients with right jaw pain (*M* = 28.02, SD = 40.51), *t*(18) = 1.13, *p* = .27. In the control group, the PSS was close to zero (*M* =  − 0.12, SD = 27.40), and did not differ from the actual point of simultaneity (zero), *t*(19) =  − 0.02, *p* = 0.98. Note that the mean PSS was not significantly larger in the TMD group than in the healthy control group, despite a medium effect size, *t*(38) = 1.59, *p* = 0.12, *d* = 0.50, 95% CI [−0.13–1.13].

The JND (about which no hypothesis was formulated) was not significantly different between the TMD group (*M* = 73.87, SD = 20.84) and the control group (*M* = 64.42, SD = 15.42), *t*(38) = 1.63, *p* = .11.

### Secondary analyses

Pearson Correlations in the TMD group are shown in Table 3. Of the 10 calculated correlations between the PSS and the questionnaire scores, most did not reach significance, except for one significant positive correlation between the PSS and the somatic focus subscale of the TSK-TMD (*r* = .50, *p* = .025), suggesting that higher scores on this scale were associated with a tactile processing bias toward the painful side of the jaw.

## Discussion

This study investigated hypervigilance for somatosensory information in TMD patients with chronic unilateral orofacial pain. Specifically, we tested the hypothesis that processing of tactile stimuli would be biased towards the painful side of the jaw in TMD patients. While the average PSS in healthy controls was approximately zero, it was positive (about 18 ms) in the TMD group, but the difference with the actual point of simultaneity (0 ms) did not reach conventional levels of statistical significance (*p* = .07), and there was also no significant difference with the control group. Strong interpretation of these results is difficult, given that confidence intervals were large, and that the study was only sufficiently powered to detect a medium effect size. It is possible that true effects are smaller than what was expected based on the study of Vanden Bulcke et al. (2013) because of several differences (left-right side of the jaw versus left-right hand; natural tonic pain versus experimentally induced anticipation of phasic pain stimuli). A follow-up study powered to detect small-to-medium effects with 90% chance is recommended before drawing strong conclusions.

Exploratory correlation analyses were conducted to gain insight in individual variability of spatial bias to the painful side of the jaw (i.e., PSS) in the TMD group. Most of the correlations between PSS and self-reported cognitive-affective variables were not significant, but we observed a significant positive association between the PSS and the “somatic focus” subscale of the TSK-TMD, suggesting that those who tend to appraise somatic sensations in their jaw in a threatening way, show a stronger spatial bias in tactile processing to the painful side of the jaw. This finding fits well with previous findings showing that levels of pain-related fear are associated with self-reported jaw activity limitations and disability in TMD patients (Turner et al., 2001; Visscher et al., 2010) and faster detection of innocuous electrical stimuli in chronic low back pain patients (Peters, Vlaeyen & Kunnen, 2002). However, the importance of this significant correlation should not be overstated, given the exploratory nature of this analysis, the small sample, and the large amount of correlations increasing the risk of type-I error.

It is interesting to compare our results with previous findings in patients with other unilateral pain complaints. Studies using tactile TOJ methodology have shown that in patients with both unilateral chronic low back pain and complex regional pain syndrome, tactile processing at the painful side appeared be attenuated (Moseley, Gallace & Spence, 2009; Moseley, Gallace & Iannetti, 2012a; Moseley, Gallagher & Gallace, 2012b). More specific, these authors found shifts in PSS so that tactile stimuli at the affected side had to be presented before stimuli at the other side, to be perceived as simultaneous. It has been argued that impaired tactile processing at the affected body part may be a consequence of disrupted spatial representation (Moseley, Gallace & Spence, 2009) or spatial “inattention” (Reid et al., 2016). Perhaps this feature is only present in specific populations of chronic pain patients, and not in (our sample of) patients with TMD. It should also be noted that there are minor methodological differences between our study and other experiments, especially regarding the SOAs used in the tactile TOJ. More specifically, we used SOAs between 10 and 200 ms, whereas in the studies of Moseley and colleagues, the range was between 10 and 240 ms. It is unlikely, though, that these small differences would be responsible for obtaining opposite effects. Nevertheless, our findings show that we should be cautious in generalizing the idea of disrupted tactile processing in painful body parts to the entire population of unilateral chronic pain patients (Van Damme et al., 2016). More research with more diverse and larger samples of chronic pain patients (e.g., Van Damme et al., 2014; Van Damme et al., 2015), allowing identification of moderating factors of enhanced versus attenuated tactile processing, is highly recommended.

Several issues deserve further discussion. First, the overall perceived intensity of tactile stimuli (across both sides) was higher in the TMD group than in the control group. This is an intriguing finding, because the objective (actual) intensity was not different between groups, and it was individually calibrated to the same rating (3 on a 1–5 rating scale) before the start of the experiment. This may suggest some form of sensitization or delayed habituation to somatosensory input in TMD patients. Note that this is not in line with previous findings of elevated tactile thresholds in these patients (Hollins & Sigurdsson, 1998; Hollins et al., 1996). Because amplified perception in the current study was not specific to the painful side, it cannot account for any spatial attention bias. Also note that in the TMD group there was no difference in tactile intensity between the painful and the non-painful side of the jaw, which is in line with previous findings (Ayesh, Jensen & Svensson, 2007; Kothari et al., 2015), although these studies did not exclusively include patients with unilateral pain, and also examined differences between the most and the least painful side of the jaw in patients with bilateral pain.

Second, the spatial bias in TMD patients was small, and individual variability was high, with secondary analyses suggesting stronger bias in those patients characterized by threatening appraisal of bodily signals in the jaw. An interesting avenue for future research would be to investigate the effect of contextual factors, such as anticipation of pain, on spatial bias. It should be noted that in this study, there was no induction of any bodily threat. It might be that situations in which patients actively anticipate pain would increase spatial bias to the painful jaw. It could be recommended that future studies examine somatosensory processing while patients are anticipating a painful dental procedure, or after experimentally inducing pain anticipation by requiring patients to perform certain activities using the jaw muscles. There have been successful attempts to implement pain-related physical activities in somatosensory attention paradigms in healthy volunteers (Clauwaert et al., in press) as well as in whiplash patients with chronic neck pain (Vangronsveld et al., 2007). Third, we should be cautious in generalizing the results to TMD patients with bilateral orofacial pain, and call for further research in these samples. Fourth, it has to be noted that the study protocol and statistical plan have not been pre-registered for peer review.

## Conclusions

Our main hypothesis, that TMD patients would be characterized by a spatial tactile bias towards the painful side of the jaw, was not statistically confirmed, although the effect was in the expected direction. Given the higher than anticipated variability in PSS, inadequate power could not be ruled out a possible reason for this lack of statistical significance. In addition, the significant positive correlation between the amount of spatial bias and the somatic focus subscale of the TSK-TMD, suggests that somatosensory attending to the painful side of the jaw might be elevated especially in patients who are characterized by fear-avoidance beliefs, but a more adequately powered study would be needed to confirm this.

##  Supplemental Information

10.7717/peerj.4310/supp-1 Data S1 TMD study raw data The file contains the raw data for all behavioral and self-report variables collected in this study. Also included is a tab in which all variable names are explained.

## Additional Information and Declarations

### Data Availability

The following information was supplied regarding data availability: The raw data has been provided as a  Supplemental File.
